# Social network, fair payment, subjective well-being, and general health: a moderation mediation analysis

**DOI:** 10.3389/fpubh.2024.1418394

**Published:** 2024-08-26

**Authors:** Abdurrahim Güler, Murat Yıldırım, Juan Gómez-Salgado

**Affiliations:** ^1^Department of Sociology, Ağrı İbrahim Çeçen University, Ağrı, Türkiye; ^2^Department of Psychology, Ağrı İbrahim Çeçen University, Ağrı, Türkiye; ^3^Graduate Studies and Research, Lebanese American University, Beirut, Lebanon; ^4^Department of Sociology, Social Work and Public Health, Faculty of Labour Sciences, University of Huelva, Huelva, Spain; ^5^Escuela de Posgrado, Universidad de Especialidades Espíritu Santo, Guayaquil, Ecuador

**Keywords:** subjective well-being, social networks, subjective general health, perception of fair payment, moderated mediation model

## Abstract

**Objective:**

This research aimed to investigate whether subjective general health mediated the relationship between social networks and subjective well-being and whether the perception of fair payment moderated the mediating effect of subjective general health on subjective well-being.

**Methods:**

Data were drawn from round 9 of the European Social Survey (ESS), involving 3,843 respondents from 19 countries, with ages ranging from 65 to 90 years (Mean_age_ = 73.88 ± 6.61 years). The participants completed self-reported measures assessing subjective well-being, social networks, subjective general health, and perception of fair payment.

**Results:**

Subjective general health played a mediating role in the relationship between social networks and subjective well-being. The perception of fair payment emerged as a moderator in the mediating effect of subjective general health on the association between social networks and subjective well-being.

**Conclusion:**

This study suggests that the impact of social networks on both subjective general health and subjective well-being is contingent upon individuals’ perceptions of fair payment. These results highlight the significance of social networks in fostering social connections and promoting overall subjective well-being.

## Introduction

The concept of subjective well-being has been discussed since the times of Aristotle and John Stuart Mill and continues to be an important topic in contemporary psychology, sociology, and related fields (1). Subjective well-being is a multidimensional construct that includes the cognitive and affective dimensions of an individual’s life, namely positive affect, negative affect, and satisfaction with life (2, 3). The affective evaluations reflect the relatively short-term situation-dependent presence of pleasant and absence of unpleasant feelings in people’s reactions to life events (4). In contrast, life satisfaction is conceptualized as the cognitive dimension of subjective well-being based on longer-term overall evaluations and beliefs of one’s life (5). The assessment can be limited to specific domains of life such as satisfaction with work, family life, income, or health (6, 7). In this respect, people can have a good life but not be satisfied with that life or can be satisfied with a not good life such as one can have a satisfying marriage but be dissatisfied with life in general (2, 8, 9).

Typically, having greater subjective well-being stands as the most important goal for many people around the globe (10). Greater subjective well-being is not only feeling better, but it also holds practical values (11). Previous research has indicated that subjective wellbeing is associated with a wide range of positive outcomes across various domains, including better physical health, stronger social relationships, higher productivity, and greater resilience to stress and adversity alongside lower maladaptive beliefs hindering positive human functioning (2, 4, 12–20). Furthermore, relevant research has provided evidence supporting the advantages of heightened subjective well-being, which include fostering fulfilling social relations promoting increased engagement in activities and having better conflict-resolution skills (11). Additionally, people with greater subjective well-being are not only healthier, but they are also more fulfilling social relationships, more likely to contribute to the communities, have longer life expectancies, and reduced divorce rates (11, 21, 22).

### Social network and subjective general health

Social networks are powerful assets of the people in one’s life that can be embodied in bonds among family, friends, neighbors, coworkers, or others (1, 23). Evidence suggests that social networks could serve a key role in facilitating improvements in individuals’ general health by their influence on subjective well-being (24). Research findings consistently reported that the size of social networks from family, community, and friends and acquaintances (25, 26), quality of relationships (27), and increased frequency of social interactions (1) yielded positive effect on subjective well-being. Further, a substantial body of research documented that people with better quality relationships, encompassing family, friends, and romantic partners tend to report greater general health, exhibit longer lifespans, and experience fewer health-related issues (28, 29). Social networks may further be more critical to subjective well-being for the older adult with lower subjective general health and less fair payment.

Subjective general health refers to a self-reported evaluation made by the person of their health status (30). In this sense, it is important to note that subjective general health may not always align with objective health measures, which are determined through tests and observations by healthcare professionals. Accordingly, the World Health Organization (WHO) characterizes general health as a ‘state of complete physical, mental, and social well-being, and not merely the absence of disease or infirmity’ (31) which implicates a broader scope than just the absence of disorders and disabilities. Aavik and Dobewall (32) posited that individuals attribute a high value to their health because valuing health could be an outcome of different motivations. In this sense, previous studies indicated that subjective general health is related to subjective well-being (33), mental health, social support (34), socioeconomic status, external resources such as education and financial status (35), and frequent social contacts (1, 36). Several studies revealed that greater subjective general health led to greater subjective well-being (37, 38) but others say the opposite when objective health status was used (39).

As existing literature showed, social network positively contributed to subjective wellbeing by providing emotional and instrumental support, companionship, and sense of belonging (25–28). Similarly, it showed that robust social network enhances individual’s subjective general health as they have resources, support systems, felt supported and been valued that help them manage health problem more effectively (31, 36–38). This enhanced subjective general health perception led to greater subjective wellbeing (33). As literature suggests, having strong social networks positively contribute to subjective general health perception, which in turn improves subjective wellbeing. Therefore, we can predict that subjective general health is a mechanism that can explain the relationship between social networks and subjective wellbeing by transmitting the benefits of social interactions into a perception of general health, which in turn enhances subjective wellbeing to be tested.

### Fair payment as a moderating

The perception of fair pay refers to the symbolic attributes of social interactions that are linked to social status (40). The perception of fair payment is grounded in objective and subjective assessments. Subjective assessment involves personal judgments about whether the compensation received aligns with effort, skills, and the value of people’s contributions to the work they have done while objective assessment refers to the extent to which one’s pay aligns with comparable rates among workers with similar skills and experience (41). The social comparison theory suggests that the perception of fairness is built on social comparison as people make judgments based on comparisons with their colleagues and similarities (42). It is important to note that economic growth was found to be not associated with greater subjective well-being when income inequality rises (43), also confirming Easterling’s work suggesting levels of subjective well-being do not tend to increase as a society becomes richer (44). A large body of research revealed that people who perceived their pay to be fair reported higher levels of subjective well-being (45, 46), links between the perception of fair payment and general health (47), and significant effects of social network in judging the perception of fair payment (48).

The rationale behind selecting the perception of fair payment as a moderating variable is to comprehend its role within the context of social networks, general health, and subjective well-being as literature found associations with both. People are not just individuals, but they live in groups that are influenced by their connections and their values (49). The social comparison theory highlights that individuals assess the fairness of their compensation by comparing it with those of others linking it with groups and social networks (42). In this vein, the research found that social connections negatively affected the perception of fairness for public sectors while positively associated with private sectors (50). Studies also found that people who perceived their pay as unfair reported adverse health outcomes independent of actual income (47, 51). Similarly, the perceptions of fair payment were found to have a significant relationship with subjective well-being among 34 countries regardless of economic growth (43). Research showed the perception of fair pay is related to the other three concepts in some ways and the association between them could be explained by the fair pay perception. However, to the best of our knowledge, it has not been formally tested whether pay fairness perceptions moderate the relationship between social networks, subjective general health, and subjective well-being. Therefore, this study aims to address this gap by examining how perceptions of pay fairness influence these relationships.

### Present study

Drawing upon the existing literature and theoretical framework presented above, the current study proposes a moderated mediation model to examine the associations between social networks, fair payment, subjective general health, and subjective well-being. Specifically, we aimed to examine whether subjective general health acts as a mediator in the relationship between social networks and subjective well-being and whether this mediation effect is influenced by the moderating variable of fair payment. In this regard, we set out to test the following hypotheses:

*Hypothesis 1*: The literature review suggests that social networks have a significant impact on subjective well-being and general health. By proposing that subjective general health mediates this relationship, we build on previous findings that link social networks with better health outcomes, which in turn are associated with greater subjective wellbeing. Subjective general health will mediate the relationship between social networks and subjective well-being, indicating that the influence of the social network on subjective well-being is partially mediated through individuals’ perceived general health status.

*Hypothesis 2*: The perception of fair payment will moderate the mediating effect of subjective general health on the relationship between social networks and subjective well-being, implying that the impact of subjective general health as a mediator may vary depending on the level of fair payment experienced by individuals within their social networks. Social comparison theory underpins this hypothesis by suggesting that perceptions of fair payment influence individuals’ wellbeing. Therefore, fair payment can enhance the positive effects of social networks on subjective wellbeing by ensuring that individuals feel adequately rewarded and supported, thereby strengthening the mediating role of subjective general health.

**Figure 1:**
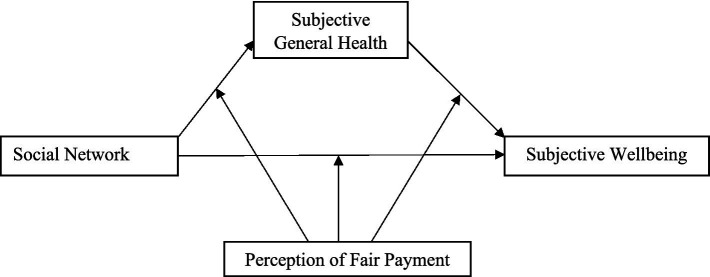
The proposed conceptual moderated mediation model.

To test these hypotheses, we constructed a moderated mediation model, as illustrated in Figure 1.

## Method

### Sample

The data used for the study was drawn from round-9 of the European Social Survey (ESS), fielded in 2019 in European countries (52). A total of 36,015 respondents were included in ESS-Round 9. After listwise deletion of missing values related to study variables, the final sample consisted of 3,843 respondents from 19 countries ranging in age from 65 to 90 with a mean and standard deviation of 73.88 and 6.61, respectively. The sample is roughly balanced in gender (45.2% men versus 54.8% women). Regarding education, 83% of the sample has some level of secondary education and above, and only 17% has less than the lower secondary level of education. The majority of the sample (60.5%) is widowed, or a civil partner died, 20.8% are legally divorced or separated, 6.8% are married or in a legally registered civil union, and 11.9% are never married single. Country samples range from 67 cases in Cyprus to 310 cases in Bulgaria. The sociodemographic characteristics of the sample and its size guarantee diversity, inclusiveness, and statistical power.

### Subjective wellbeing

Subjective wellbeing was measured with happiness and satisfaction with life. Happiness measures one’s current feelings such as emotional responses, while satisfaction with life measures one’s assessment of overall life satisfaction such as cognitive responses. A composite index constructed with the European Social Survey (52) items to measure subjective wellbeing. Happiness was measured with a single item, “Taking all things together, how happy would you say you are?.” This item was answered on an 11-point Likert type scale ranging from 0 (extremely unhappy) to 10 (extremely happy) scale. Life satisfaction was also assessed with a single item, “All things considered, how satisfied are you with your life as a whole nowadays?.” This item was rated on an 11-point Likert type scale ranging from 0 (extremely dissatisfied) to 10 (extremely satisfied) scale. We then summed the total scores of happiness and life satisfaction to create a composite score for subjective wellbeing. This approach is quite common and practical in the literature. Single-item scales showed psychometrically sound instruments for assessing subjective wellbeing indicators as they performed well compared to the multiple items (53, 54). In this study, the mean score of subjective wellbeing is obtained as 6.69, with a standard deviation of 2.14. The higher scores on this scale represent the greater subjective wellbeing.

### Social network

In this study, we considered three domains of social network (namely social support, romantic relationship, and social contacts) and each domain was measured with a single item (52). Then we combined these three questions into a single composite score to create a social network index. The first domain of social network index captured the number of people getting social support from friends, family and significant others. Respondents were asked “how many people, if any, are there with whom you can discuss intimate and personal matters.” Responses to this question were coded on a 7-point Likert type scale ranging from 0 to 6, where 0 means ‘none, 1 means ‘one person,’ 2 means ‘two people,’ 3 means ‘three people,’ 4 means ‘four-to-six people,’ 5 means ‘seven-to-nine people,’ and 6 means ‘10 or more people’. If participants had indicated none, one, and/or two people, they were coded 0 (*low social support*); otherwise, they were coded 1 (*high social support*). The second domain of social network index captured frequency of social contact. Respondents were asked, “How often do they socially meet with friends, relatives, or work colleagues.” Responses to this question were coded on a 7-point Likert type scale ranging of 1 to 7, where 1 means ‘never,’ 2 means ‘less than once a month,’ 3 means ‘once a month,’ 4 means ‘several times a month,’ 5 means ‘once a week,’ 6 means ‘several times a week,’ 7 means ‘every day.’ If participants had indicated never, less than once a month, once a month, and/or several times a month, they were coded 0 (*low social contact*); otherwise, they were coded 1 (*high social contact*). The third domain of social network index captured simply whether the respondent is currently having a romantic relationship or not. If participants had indicated yes, they were coded 1 (*had romantic relationship*); otherwise, they were coded 0 (*no romantic relationship*). To measure the social network index, we summed the standardized scores on social support, social contact, and romantic relationship questions and then divide by three, using following the formula: *social network index = (social support + social contact + romantic relationship)/3*. The possible social network scores ranged from 0 to 1, where higher scores indicate greater social network in this study.

### Subjective general health

Subjective general health was measured with a single construed item that respondent was asked to indicate their general health condition as “How is your health in general?,” with responses rated on a 5-point Likert type scale ranging from 1 (*very good*) to 5 (*very bad*) (52). The item was reverse-coded. Higher scores indicate better subjective general health.

### Perception of fair payment

The perception of fair payment was measured on a 2-item scale, using data from the European Social Survey. Respondents were asked to rate the fairness of their own gross incomes, as well as the fairness of benefits on a 9-point scale ranging from extremely unfairly too low (−4) to extremely unfairly too high (+4) (52). ESS asked questions “Would you say your gross pay is unfairly low, fair, or unfairly high?” and “Would you say your net pay/pensions/social benefits are unfairly low, fair, or unfairly high?” to capture fair payment. The mean perception of fair payment in this study was −1.47 showing people, on average assessed their income and benefits as somewhat lower than they considered fair. Social justice literature frequently employs questions like this to assess people’s evaluations of ‘expressed fairness’ (55).

### Data analysis

In the initial stages of the analysis, we conducted preliminary examinations to assess the observed scale characteristics, the assumptions required for subsequent analyses, and the relationships among the study variables. To examine the assumption of normality, we employed skewness and kurtosis values along with established cut points. Subsequently, Pearson correlation analysis was utilized to explore the correlations between the variables. Following the preliminary analyses, we tested the assumptions for the main analyses such as multicollinearity, linearity, and normality of variables and there were no violations regarding these assumptions. Afterwards, we conducted mediation and moderated mediation models using the PROCESS macro, specifically Model 4 for mediation and Model 59 for moderated mediation (56), in SPSS version 26. In the first step, we examined the mediating role of subjective general health in the relationship between social networks and subjective well-being. Next, we explored the moderating effect of the perception of fair payment on the mediating role of subjective general health in the link between social networks and subjective well-being. The interpretation of the mediation and moderated mediation models was based on standardized path estimates (*β*) and squared-multiple correlations (*R^2^*). To assess the significance of indirect effects, we employed the bootstrap method with 5,000 resamples to estimate 95% confidence intervals (CI). All analyses were performed using SPSS version 25 for Windows.

## Results

Descriptive statistics (e.g., mean, standard deviation, skewness, and kurtosis) and correlational coefficients were computed. The skewness values ranged from −0.92 to −0.03, while the kurtosis values ranged from −0.91 to 0.69, suggesting no violation regarding the normal distribution of the variables of this study.

Correlation analysis demonstrated that social networks had a significant positive correlation with the perception of fair payment (*r* = 0.20, *p* < 0.001), subjective well-being (*r* = 0.33, *p* < 0.001), and subjective general health (*r* = 0.28, *p* < 0.001). The perception of fair payment also had a significant positive correlation with subjective well-being (*r* = 0.35, *p* < 0.001) and subjective general health (*r* = 0.27, *p* < 0.001). Subjective well-being also significantly correlated with subjective general health (*r* = 0.42, *p* < 0.001). Descriptive statistics and correlations are presented in Table 1.

**Table 1:** Descriptive statistics and correlation results.

Variable	Descriptive statistics				Correlations			
	*M*	*SD*	Skew.	Kurt.	1	2	3	4
1. Social network	0.41	0.31	0.20	−0.91	–	0.20**	0.33**	0.28**
2. Perception of fair payment	−1.47	1.56	−0.03	−0.51		–	0.35**	0.27**
3. Subjective wellbeing	6.69	2.14	−0.92	0.69			–	0.42**
4. Subjective general health	3.33	0.91	−0.25	−0.01				–

**p < 0.001.

**Figure 2:**
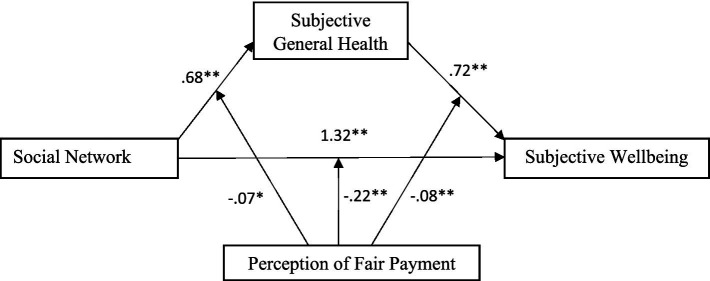
The results of moderated mediation model.

The mediating role of subjective general health on the relationship between social networks and subjective well-being was examined and the moderating effect of the perception of fair payment on the mediating role of subjective general health in this association was tested using conditional process analysis as presented in Figure 2. Findings from mediation analysis using PROCESS macro-Model 4 showed that social network was a significant positive predictor of subjective general health (*b* = 0.68, *t* = 14.97, *p* < 0.001) and subjective wellbeing (*b* = 1.32, *t* = 13.58, *p* < 0.001). The perception of fair payment significantly positively predicted subjective general health (*b* = 0.13, *t* = 13.84, *p* < 0.001) and subjective well-being (*b* = 0.33, *t* = 16.65, *p* < 0.001). Subjective general health was a significant predictor of subjective well-being (*b* = 0.72, *t* = 21.45, *p* < 0.001). Further, the interactions between the social network and subjective well-being; between social network and subjective general health; and between subjective general health and subjective well-being were negative and significant, explaining %2, %3, and %1 of variances in the associations, respectively, as seen in Table 2.

**Table 2:** The moderated mediation model analysis.

Antecedent	Consequent							
	M (Subjective general health)				Y (Subjective wellbeing)			
	Coeff	*SE*	*t*	*p*	Coeff	*SE*	*t*	*p*
X(Social network)	0.068	0.046	14.969	<0.001	1.323	0.097	13.575	<0.001
W(Fair payment)	0.127	0.009	13.837	<0.001	0.325	0.020	16.653	<0.001
X*W (Social network* Fair payment)	−0.066	0.030	−2.227	<0.05				
M (Subjective general health)					0.720	0.034	21.445	<0.001
M*W (Subjective general health* Fair payment)					−0.083	0.021	−4.054	<0.001
W*X(Fair payment* Social network)					−0.217	0.064	−3.361	<0.001
Observations	3,843				3,843			
*R* ^2^	0.121				0.294			
*R*	0.348				0.543			
*F* statistic	*F*(3, 3,839) = 176.200, *p* < 0.001				*F*(3, 3,837) = 320.190, *p* < 0.001			

Conditional effects of social network on subjective well-being					
Fair payment		Effect	SE	BootLLCI	BootULCI
Direct effect	*M*_ean_ − 1 SD	1.661	0.143	1.381	1.941
	*M*_ean_	1.323	0.097	1.132	1.514
	*M*_ean_ + 1 SD	0.985	0.137	0.715	1.254

		Effect	BootSE	BootLLCI	BootULCI
Indirect effect	*M*_ean_ − 1 SD	0.667	0.072	0.528	0.812
	*M*_ean_	0.490	0.041	0.409	0.573
	*M*_ean_ + 1 SD	0.341	0.050	0.245	0.444

SE, standard error; Coeff, unstandardized coefficient; X, independent variable; M, mediator variable; W, moderator variable; Y, dependent variable; SD, standard deviation.

The moderated mediation model of this study was tested using PROCESS macro (Model 59) which assumes that a simple mediation model with all three paths moderated by a common moderator. The results showed that the perception of fair payment had significantly negatively moderating effect between the social network and subjective general health (*b* = −0.066, *t* = −2.227, *p* < 0.05), with 95% CI [−0.124, −0.008], between subjective general health and subjective wellbeing (*b* = −0.083, *t* = −4.054, *p* < 0.01), with 95% CI [−0.124, −0.043], and between social network and subjective wellbeing (*b* = −0.217, *t* = −3.361, *p* < 0.01), with 95% CI [−0.343, −0.090]. These results suggest that the perception of fair payment can play a negative moderating role in predicting subjective general health by social networks and predicting subjective well-being by subjective general health and social networks.

**Figure 3:**
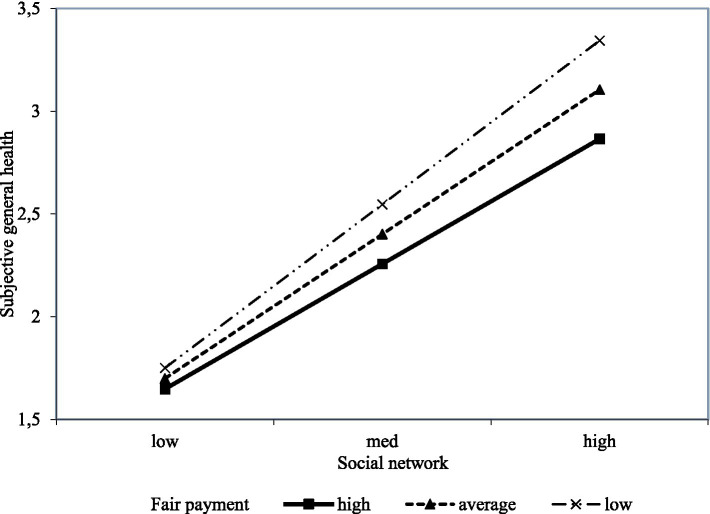
Fair payment moderates social networks and subjective general health.

To better understand how fair payment moderates the relationship between social networks and subjective general health, the perception of fair payment was divided into unfairly low, average, and unfairly high groups by *M* ± 1 SD, and three simple slope tests were performed. Results of the first simple slope plot indicated that the social network of individuals with unfairly low payment is a much stronger predictor of subjective general health than individuals with average and unfairly high payment. Specifically, as Figure 3 showed a steeper gradient for unfairly lower payment, and for individuals with unfairly low payment (M − 1 SD), social networks had a significant negative predictive effect on subjective general health. As the level of payment increased, the strength of the relationship between social networks and subjective general health decreased.

**Figure 4:**
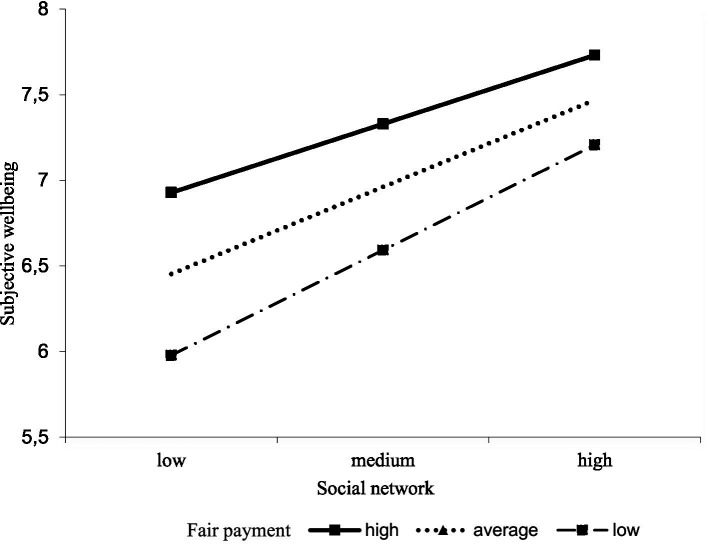
The perception of fair payment moderates social networks and subjective well-being.

The second simple slope plot indicated that the social network of individuals with unfairly low payment is a much stronger predictor of subjective well-being than individuals with average and unfairly high payment. As Figure 4 shows, the impacts of social networks on subjective well-being are much stronger at unfairly lower payment. However, for individuals with an unfairly higher payment, the line tends to straighten, and the impact of the increase in social networks on subjective well-being was weakened. As the level of payment increased, the strength of the relationship between social networks and subjective general health decreased.

**Figure 5:**
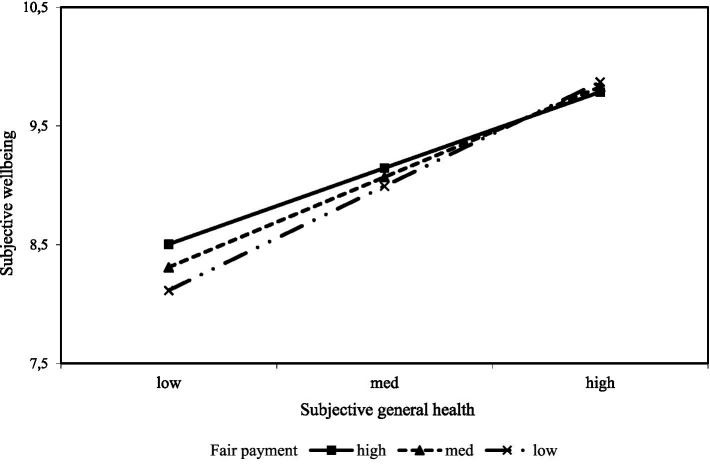
The perception of fair payment moderates subjective general health and subjective well-being.

The third simple slope plot indicated that the subjective general health of individuals with unfairly low payment is a slightly stronger predictor of subjective wellbeing than individuals with average and unfairly high payment. As Figure 5 shows, the impact of subjective general health on subjective well-being is stronger at unfairly lower payment. However, for individuals with an unfairly higher payment, the line tends to straighten, and the impact of the increase in subjective general health on subjective well-being weakened. As the level of payment increased, the strength of the relationship between subjective general health and subjective general health decreased.

The conditional indirect effect indicated that the indirect effect is high at unfairly low payment, reduced at average payment, and further reduced at unfairly low payment. However, the conditional indirect effects are significant at all three levels. The results suggested that the indirect effect of social networks on subjective well-being through subjective general health moderated by the perception of fair payment is significant.

## Discussion

The present study delved into the intricate relationships among social networks, the perception of fair payment, subjective general health, and subjective well-being. The aim was to unravel how these factors interplay and mutually influence each other, while also shedding light on the moderating impact of the perception of fair payment and the mediating role of subjective general health in shaping overall subjective well-being.

The correlation analysis yielded significant positive associations between social networks, the perception of fair payment, subjective general health, and subjective well-being. These findings align with prior research (33, 39, 46, 47), underscoring the interconnected nature of these variables. The results suggest that individuals with more social networks tend to view their remuneration as fair, experience improved subjective general health, and report higher levels of subjective well-being. This coherence resonates with earlier studies emphasizing the pivotal role of social connections in fostering positive well-being and physical health outcomes (26, 27). However, it’s important to acknowledge that the study did not categorize respondents based on the strength of their social relationships, which restricts comparisons between the effects of strong versus weak relationships.

Moving beyond correlation, the mediation and moderation analyses provided deeper insights into the underlying mechanisms governing the relationship between social networks and subjective well-being. The results of the mediation analysis provide evidence for the initial hypothesis by demonstrating that subjective general health acts as a mediator between social networks and subjective well-being. Consistent with previous studies (24, 29, 35, 37), which emphasize that not only do social networks directly contribute to subjective well-being, but they also exert their influence indirectly through enhanced subjective general health. This mediating mechanism underscores the pivotal role of extensive social networks in fostering better health outcomes.

Furthermore, one focal point of the study was the moderating role of the perception of fair payment among social networks, subjective general health, and subjective well-being. The findings revealed that the perception of fair payment serves a significant moderating role in the associations between social networks, subjective general health, and subjective well-being, thereby affirming the study’s second hypothesis. By categorizing the perception of fair payment into distinct groups, a nuanced exploration of these effects became possible. Notably, individuals receiving unfairly low payment exhibited heightened sensitivity to the impact of social networks on subjective general health and subjective well-being. This suggests that the impact of social networks on both subjective general health and well-being is contingent upon individuals’ perceptions of payment fairness. The findings suggest that when individuals perceive their compensation as unjustly low, the effects of their social networks on subjective health and well-being become more pronounced, possibly due to feelings of inequality or discontent (23, 28). The findings also suggest that when individuals perceive their payments as unfairly low, the effects of better subjective general health on subjective well-being tend to be greater (32, 38, 47).

Potential reasons behind the relationship are that the perception of fair payment could significantly influence an individual’s sense of self-worth, which in turn affects their subjective wellbeing. In this sense, the fairness of pay can buffer against the stress and dissatisfaction that may arise from less social interactions, thereby enhancing the positive impact of social networks on subjective wellbeing. Moreover, the perception of fair payment may affect individuals’ engagement with their social networks. Fairly compensated individuals might be more inclined to participate in social activities and maintain robust social connections, as they may have more resources. Conversely, individuals who perceive their payment as unfair may experience feelings of injustice and resentment, which can heighten their sensitivity to social interactions and exacerbate negative health outcomes. These negative emotions can create a feedback loop, where the stress and dissatisfaction from perceived unfairness further deteriorate their subjective general health, thus intensifying the negative impact on their overall subjective wellbeing. Understanding these nuanced mechanisms highlights the importance of addressing perceptions of fair payment to foster a healthier and more supportive social environment that enhances overall subjective wellbeing.

Although the present findings contribute to the literature by examining the relationships between subjective general health, social networks, and subjective well-being forward in some important respects, the study has several limitations that warrant consideration. Data relied on self-report measures, which can introduce bias, such as social desirability bias or recall bias, which may affect the accuracy of the results. Future studies could address this limitation by incorporating objective measures or third-party reports to validate self-reported data. Additionally, the study’s cross-sectional design, in which the data is collected at one point in time, restricts the ability to infer causality conclusively; hence, it is impossible to establish causal relationships between variables. Longitudinal studies would be more effective in establishing causal relationships. In the future, research on subjective well-being should concentrate on formulating more sophisticated measures that capture the multidimensionality of the concept. Furthermore, exploring the contribution of other potential mediators and moderators, such as coping mechanisms or job satisfaction, could enrich the understanding of these associations. A final limitation of this study is the omission of important demographic variables such as age, gender, income, and psychological traits which could have influenced the connections between social networks, health, and happiness. These constructs were not fully considered in our analysis to avoid the complexity of the analysis. Future studies, diverse population samples, and intervention research could deepen our understanding and guide effective strategies to enhance well-being. Exploring different cultural contexts would also be useful to improve the generalizability of the findings.

Despite these limitations, the findings of this study offer several implications for both policymakers and practitioners. Firstly, the findings highlight the significance of social networks in fostering social connections and promoting perceptions of fair payment and overall subjective well-being. This strengthens the idea that individuals’ social environments significantly impact their holistic health. Policymakers and practitioners could consider and facilitate allocating resources to programs that promote social connection within communities and combat social isolation in older adults such as community programs, senior centers with social events, volunteer opportunities connecting people with similar interest, or workplace initiatives that promote social interaction and connect them with other people. Policymakers could explore ways to utilize technology to connect older adults with social networks such as offering training on video conferencing platforms and providing access to age-friendly online communities. This study further emphasizes the importance of fair pay in shaping subjective wellbeing. Policymakers could use these findings to support more stringent regulations or policies promoting fair compensation practices. Secondly, it should be noted that there is a strong association between subjective general health and greater subjective well-being, which holds true regardless of whether respondents had chronic medical conditions or were from the general population. Therefore, policymakers should prioritize improving the health status of the general population rather than solely focusing on improving the health of individuals with chronic medical conditions to promote subjective well-being. Additionally, the recognition of the moderating effect of perceived fair payment offers insights for organizations and policymakers. Organizations could promote a culture of fair pay and work conditions that may enhance not only subjective well-being but also boost the beneficial impacts of social networks on health outcomes. Finally, healthcare professionals could consider integrating strategies to encourage social interaction alongside conventional health promotion efforts, particularly for those who see their compensation as unjust.

Observed results suggest promise for interventions targeting social networks and perception of fair payment to improve subjective wellbeing. Programs, such as community centers offering social activities or online platforms fostering social connections utilizing technology, align with our findings on the importance of social networks. Similarly, policies promoting fair pay, including minimum wage increases or pay transparency measures, could address concerns identified in our study and potentially enhance subjective wellbeing. Future research directly testing the effectiveness of such interventions on subjective wellbeing is warranted.

In conclusion, this study offers comprehensive insights into the intricate interplay between social networks, the perception of fair payment, subjective general health, and subjective well-being. The findings underscore the importance of comprehensive social networks not only for direct contributions to subjective well-being but also for their role in enhancing subjective general health. Additionally, the study illuminates how perceptions of payment fairness can magnify the impact of social networks on health and well-being, highlighting the intricate connections between these factors. By revealing the mediating and moderating mechanisms, the study advances our understanding of how social factors interact to shape individual experiences in contemporary society. This discussion will delve into the implications of these findings and their contributions to the existing literature.

## Data Availability

The datasets presented in this study can be found in online repositories. The names of the repository/repositories and accession number(s) can be found at: https://ess-search.nsd.no/en/study/bdc7c350-1029-4cb3-9d5e-53f668b8fa74.
